# Enhancing integration for greater sustainability of HIV, TB and malaria responses: insights and lessons from African community-led monitoring programs

**DOI:** 10.3389/fpubh.2026.1902636

**Published:** 2026-10-14

**Authors:** Gemma Oberth, Jelena Bozinovski, Susan Perez

**Affiliations:** 1 Center for Social Science Research, University of Cape Town, Cape Town, South Africa; 2 International Treatment Preparedness Coalition, Johannesburg, South Africa; 3 The Global Fund to Fight AIDS, Tuberculosis and Malaria, Community, Rights and Gender Department, Geneva, Switzerland

**Keywords:** community engagement, community-led monitoring, HIV, integration, malaria, sustainability, TB

## Abstract

Global HIV, TB and malaria responses have achieved substantial gains, largely through vertical programs and parallel systems. With shrinking external funding, integration is a strategic process that supports sustainability by replacing fragmented approaches with unified models, embedded in public health systems. Community-led monitoring (CLM) provides community-generated evidence that can inform efforts to advance the integration agenda. This community case study synthesizes evidence and lessons from secondary CLM reports covering more than 4,000 health facilities in 16 African countries, according to integration focus areas for the Global Fund to Fight AIDS, Tuberculosis and Malaria. At the service level, CLM identifies persistent fragmentation, including gaps in TB/HIV co-infection services, weak coordination between programs, and patient pathways requiring multiple visits. CLM data have been used to advocate for integrated solutions, including same-day service delivery and stronger linkages between HIV, sexual and reproductive health, gender-based violence, and mental health services. CLM also provides insights into the integration of vertical programs into primary health care, particularly during donor transitions. At the systems level, CLM is being used within efforts to strengthen integration, including through unified monitoring frameworks, incorporation of community data into national systems, and improvements in supply chains, laboratories and workforce capacity. CLM data have been used to inform advocacy and decision-making related to integrated sample transportations systems, and expansion of social health insurance coverage. Key lessons highlight the importance of integrated monitoring tools, the role of community actors in facilitating integration, and the potential for CLM to contribute to broader health system reform processes.

## 1 Introduction

Global HIV, TB and malaria responses have achieved substantial gains, largely through vertical, donor-funded programs with disease-specific architecture that operates parallel to primary health care systems (1–4). With shrinking external funding, integration is a strategic process that supports the sustainability of HIV, TB and malaria responses by replacing fragmented approaches with unified models, embedded in public health systems (5–10).

Integration contributes to the sustainability of HIV, TB and malaria responses by enhancing the efficiency of service delivery, improving disease outcomes, and strengthening health system resilience (11, 12). For example, integrated care for HIV, diabetes and hypertension could reduce costs from $18.67 to $5.86 per patient visit in Uganda and Tanzania (13). Integrating pediatric TB into child healthcare services would increase treatment success by 5% in Cameroon and 9% in Kenya (14). Integrated community case management for malaria was resilient during active conflict in South Sudan, and strengthened health systems in Ethiopia, Malawi and Mozambique (15, 16).

Community engagement is vital part of the integration process (17). Integration should be co-led with communities affected by the three diseases, including people living with HIV, TB survivors, sex workers, men who have sex with men, people who use drugs, transgender people, prisoners, refugees, mineworkers, mobile populations, and others (12, 18).

Community-led monitoring (CLM) is an intervention which empowers recipients of care to take a leading role in shaping health responses (19–23). It involves the systematic collection and analysis of data on health service delivery by trained peer monitors, which is then rapidly fed back to decision-makers (24–26). CLM provides a unique opportunity to advance the integration agenda while ensuring meaningful community engagement (27).

## 2 Context

This case study captures lessons learned on HIV, TB and malaria integration from CLM programs in 16 African countries, implemented in more than 4,000 health facilities covering 55% of health districts and a range of health issues (Table 1). These 16 countries were selected for the case study because they are priority CLM countries for the Global Fund to Fight AIDS, Tuberculosis and Malaria (the Global Fund) and are receiving dedicated technical assistance. The Global Fund invests nearly US$ 42 million in these 16 CLM programs during Grant Cycle 7 (2023–2025 allocation period), complimented by support from other partners (28).

**Table 1:** Overview of community-led monitoring programs in 16 African countries (as of Dec. 2024).

Country	Services monitored	Facilities monitored	Geographic coverage*
Angola	HIV, TB, malaria, SRH, HPV, human rights	22	19%
Burkina Faso	HIV, TB, malaria, vaccination	1,175	86%
DRC	HIV, TB, malaria, Mpox, cholera	733	19%
Ethiopia	HV, sexual and reproductive health (SRH), mental health	87	11%
Ghana	HIV, TB, malaria	100	18%
Guinea	HIV, TB, malaria, vaccination	75	100%
Lesotho	HIV, TB, SRH, maternal health, cancer, gender-based violence (GBV)	194	100%
Liberia	HIV, TB, malaria	17	20%
Malawi	HIV, TB, SRH, nutrition, GBV	174	86%
Mali	HIV, malaria, GBV, RMNCAH	39	45%
Mauritius	HIV, nutrition	8	67%
Namibia	HIV, TB, malaria, SRH, vaccination, cancer, diabetes, hypertension, epilepsy	106	87%
Nigeria	HIV, TB, malaria, SRH, GBV, pandemic preparedness	382	100%
Rwanda	HIV, TB, malaria, SRH, GBV	20	10%
South Africa	HIV, TB, hepatitis, mental health, SRH, cancer, GBV, substance use	810	81%
Zimbabwe	HIV, TB, malaria, SRH, non-communicable diseases	252	33%

*Geographic coverage is estimated based on the number of districts (or equivalent sub-national health units) with a CLM mechanism in place, divided by the total number of districts in the country.

A case study methodology was chosen to reflect upon CLM as a relatively novel intervention that is geared towards improving health services and policy for affected communities. Indeed, CLM for HIV, TB and malaria responses was formally established and scaled in the past 5 years (29). Community accountability and treatment literacy movements have historically contributed to similar monitoring functions (30). To date, CLM has largely been studied in terms of its contribution to the availability, accessibility, acceptability and quality of specific services (31–36). Little is known about how CLM promotes HIV, TB and malaria integration.

While integration as a concept is not new, it has received renewed attention in global health due to the changing health financing landscape and the urgent need to prioritize limited resources. At the Global Fund’s February 2026 Board meeting, integration was introduced as a strategic shift for its eighth grant cycle (GC8) 2026–2028 (12). This case study helps fill a knowledge gap in how CLM contributes to this new strategic shift.

For this case study, CLM programs were assessed for their contribution to: (1) service delivery integration, and (2) health systems integration, using the Global Fund’s integration guidance as the methodological framework to inform the synthesis (12). This includes the service delivery points, systems components, and prioritization considerations listed in the guidance.

Conceptually, the Global Fund considers integrated service delivery and systems for health to contribute to improved health outcomes and increased efficiency and cost-savings, underpinned by principles such as ownership, differentiation, partnership, and community engagement (Figure 1).

**Figure 1:**
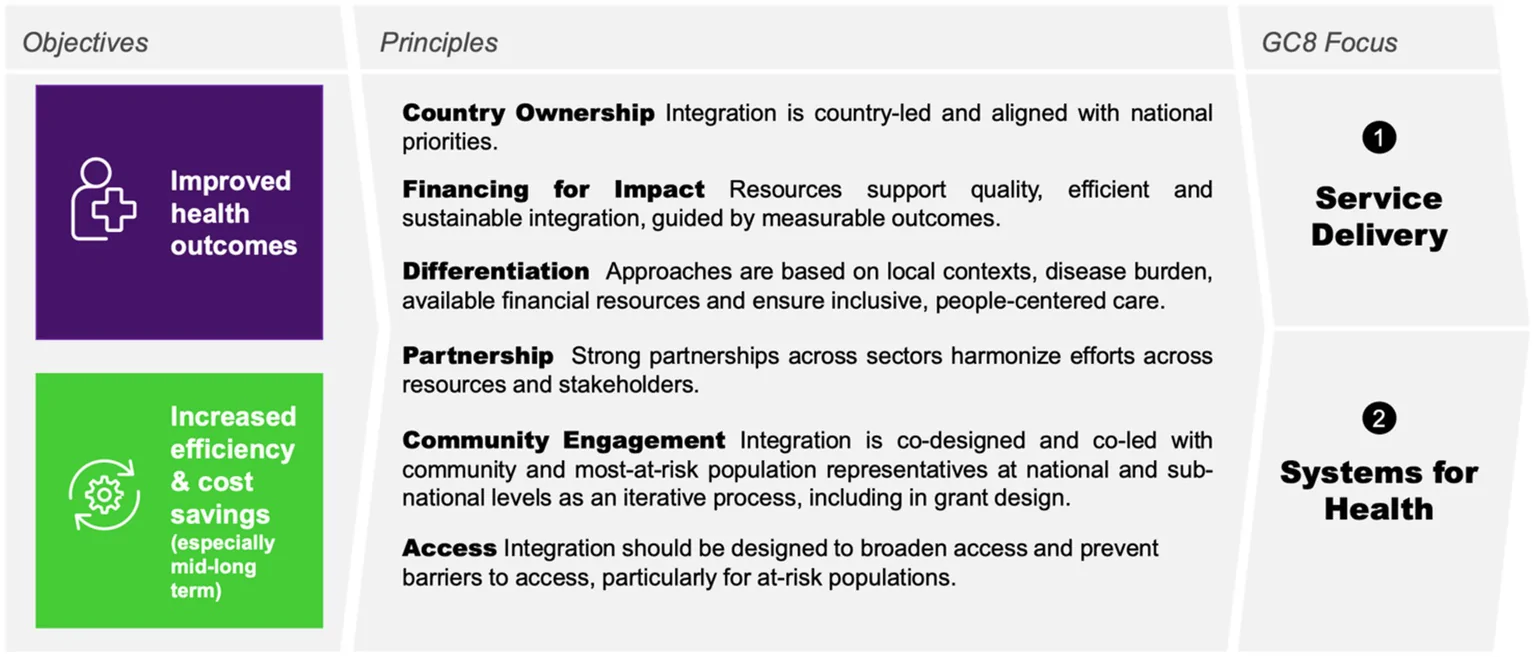
Objectives, principles and focus for enhancing integration in GC8 (12).

Data were collected from secondary sources, published online or obtained directly from CLM implementers. The document identification process was purposive rather than systematic, with the aim of capturing available CLM evidence relevant to HIV, TB and malaria integration across the 16 focus countries. Reports were included if they were specific to HIV, TB and/or malaria; from one of the 16 focus countries; included CLM data; and linked to at least one integration priority in the Global Fund’s guidance. CLM documentation from Global Fund-supported programs as well as other partners were included in the case study. Where possible, evidence from CLM implementer reports was triangulated with other sources—such as peer-reviewed literature and reports from national health authorities—for quality assurance and to limit reporting bias. Based on this criteria, this case study includes 22 publications from CLM implementers, 11 peer-reviewed CLM publications, and 8 publications from national health authorities. Documents were analyzed using a deductive thematic approach, with relevant findings extracted, categorized, and synthesized according to an *a priori* framework comprising the service delivery and health systems integration domains and associated priority areas set out in the Global Fund’s integration guidance.

Given that no human participants were involved in the production of this case study, and ethical approvals were obtained in the respective countries, the University of Cape Town’s Center for Social Science Research (CSSR) Research Ethics Committee determined that additional ethical review for this case study was not required (Letter dated 22 June 2026).

## 3 Results

### 3.1 The contribution of CLM to service delivery integration

#### 3.1.1 Integrating HIV, TB and malaria services

Given the prevalence of HIV, TB and malaria—and high co-infection/co-occurrence rates—in Africa, integrated prevention and treatment services of these three diseases is essential (37–39).

In Guinea, CLM detected that 64 out of 184 TB patients at 9 out of 15 monitored sites had not received an HIV test in the first quarter of 2024 (34.8%), including 35 who said the test had not been offered (40). Data indicate limited provider capacity in integrated TB/HIV care and shortages of HIV testing inputs. Following CLM advocacy with facility managers, by the second quarter of 2025, reports described proactive management of small-scale stockouts (41). Yet, CLM reports show inconsistent HIV testing among surveyed TB patients, fluctuating from 65.2% in Q1 2024, to 78.5% in Q3 2024, 80.3% in Q4 2024, 70.9% in Q1 2025 and 79.5% in Q2 2025 (40–44). This suggests the need for sustained advocacy and ongoing problem-solving to address recurring barriers and consolidate progress in integrating TB and HIV services.

#### 3.1.2 Integrating HIV, TB and malaria with other disease responses

Guidance suggests that HIV, TB and malaria should be further integrated with other health responses, including non-communicable diseases and sexual and reproductive health (17).

In Angola, CLM monitors the integration of HIV and SRH services for young people aged 10–24 years. In 2024, among 106 respondents, 16% in Benguela and 24% in Luanda report needing multiple appointments and multiple visits to receive their desired service package (45). Implementers are engaging facility staff to ensure that youth-friendly services are scheduled on the same day. Further, in 2026, Angola began monitoring access to HPV (genital warts) services for HIV key populations, including men who have sex with men. Results will inform actions to strengthen integrated HIV and STI service delivery.

In South Africa (West Rand District), CLM detected an association between HIV testing and contraceptive service uptake among adolescent girls and young women aged 15–24 years (*r* = 0.36, *p* < 0.001). CLM implementers began advocating for HIV and family planning service integration at facility level. One year later, this efficiency contributed to a reduction in the unit cost of finding and diagnosing one young woman living with HIV by nearly one third (31.3%) compared to non-CLM sites (46). However, at an estimated $2,852 per diagnosis, further integration and more targeted testing may be required to reach cost-effectiveness thresholds (47).

In Lesotho, CLM monitors the integration of HIV and gender-based violence services. From April to June 2024, out of 335 adolescent girls and young women living with HIV, only 133 (40%) were screened for gender-based violence and 110 were referred for GBV services (48). CLM implementers are using this data to advocate for improved referrals and linkages between HIV and GBV services (i.e., establishing standard operating procedures), in line with the National HIV and AIDS Strategic Plan 2023–2028 (49). These actions appear to have advanced national policy. In April 2026, Lesotho launched an integrated Investment Case for Maternal, Newborn and Child Health, Family Planning, Gender-Based Violence and Child Marriage (50).

In Ethiopia, where HIV-related stigma is very high, the CLM program recognizes the importance of mental health integration in service uptake and improvement. Networks of young people living with HIV are involved in CLM implementation, and focus specifically on addressing the issue:

*“As positive youth, we support children who know their positive status, who will be depressed, feel bad, lose confidence, and go through self-isolation […]. But after interacting with us they completely changed, accept their status and adhere to follow-up and build confidence to live. This is what communities can change”* (51).

Mental health is also integrated into Nigeria’s CLM program. The data show that 8,459 people needed but could not access mental health services in the first half of 2024, including nearly half of people living with HIV (48%), youth (47%) and key populations (such as sex workers, men who have sex with men, transgender people and people who use drugs; 44%) (52).

#### 3.1.3 Integrating parallel HIV, TB and malaria services into primary health care

It is recommended that parallel HIV, TB and malaria services are integrated within national health systems, with particular care taken for key populations (53, 54).

In South Africa, CLM tracked the linkage of 1,193 HIV key populations to public and private health facilities following the closure of USAID-supported clinics in early 2025. Over an eight-week period, 277 were successfully linked, 298 preferred to access services at private clinics, 78 refused to be linked, and 24 remained indecisive (Figure 2) (55). These data provide critical insight into the successes and challenges of integrating specialized key population services into the public health system, including those who may be lost to care.

**Figure 2:**
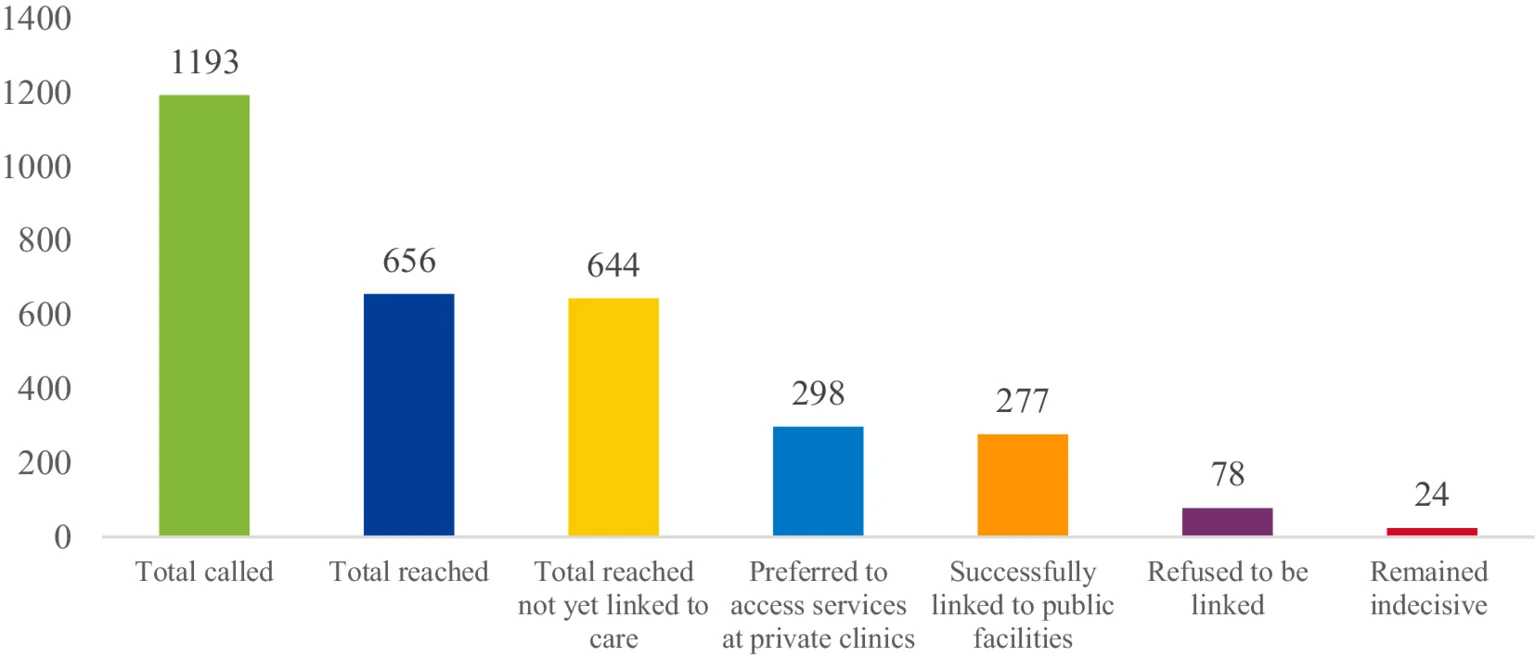
Number of HIV key populations integrated into public and private care in South Africa following the closure of USAID-supported clinics in 2025, CLM data (55).

In Malawi, CLM is tracking silent transfers from donor-funded clinics—many of which have closed (56)—to public facilities using baseline viral load suppression among new ART clients as a proxy (57). From November 2025 to January 2026, 391 new ART clients at 18 clinics in Mulanje, Phalombe and Zomba had suppressed viral loads upon presenting, suggesting some recipients of care are migrating to new facilities following the closure of US-supported sites (58). However, 23.0% of people said they are not receiving clear information on where to go to obtain HIV services that they used to access from USAID-supported clinics.

In Lesotho, findings from 9,655 CLM interviews in 2023 indicate that adolescent girls and young women living with HIV often feel uncomfortable accessing health facilities due to being segregated from other service users, which exposes them to discrimination and risks disclosing their HIV status (59). Integration within primary care reduces stigma compared to siloed HIV services, though some still prefer them for their specialized offering (60).

#### 3.1.4 Integrating HIV, TB and malaria responses into other non-health sectors

Multisectoral collaboration is identified as a key priority in HIV, TB and malaria responses (61–64). Several CLM programs are fostering engagement in HIV, TB and malaria responses beyond Ministries of Health.

A common nexus is with justice and correctional sectors. In Lesotho and Malawi, CLM is taking place in prisons, working closely with Correctional Services. In Mali, CLM implementers detected 732 human rights violations among HIV key populations between June 2023 and December 2024. CLM implementers provided integrated linkage to medical care as well as legal support for the reported cases (65). In Angola, CLM implementer are working with the Ministry of Justice to build capacity based on their human rights data.

In South Africa, data on substance use and gender-based violence are collected from the Department of Social Development and the South African Police Service (66). As of February 2022, these data are shared with the Department of Health in certain districts, following an agreement initiated through CLM (67). This data sharing agreement may help improve District Health Service recordkeeping of intersecting social and structural drivers of HIV and TB.

In Mauritius, CLM monitors the housing status of survey respondents, which is especially relevant given the program’s focus on people who use drugs. Advocacy with the Ministry of Housing and Lands is embedded in the program.

### 3.2 The contribution of CLM to health systems integration

#### 3.2.1 Integrated monitoring and evaluation systems, including community data

Eight countries (Angola, Burkina Faso, DRC, Guinea, Namibia, Nigeria, Rwanda and Zimbabwe) conduct integrated monitoring of HIV, TB and malaria, reducing vertical data collection and analysis of single diseases. In Nigeria, there is an integrated CLM indicator framework for HIV, TB and malaria, one consolidated data collection tool, and one integrated CLM data warehouse and interoperability layer with DHIS2 and the National Data Repository (68).

The integration of community-generated data into national routine program monitoring systems, including health management information systems (HMIS), is recommended for more holistic and informed decision-making (61, 69).

In DRC, CLM data is systematically included in the annual national program reviews for HIV, TB and malaria. For instance, CLM data on stockouts of malaria commodities was included in the 2024 Malaria Program Review (Figure 3) (70). The data show that around 35% of health facilities and 43% of community services experiencing monthly shortages of malaria supplies—particularly in Kongo Central—affecting key commodities such as pediatric artemisinin-based combination therapies, amoxicillin, oral rehydration salts, zinc supplements, and rapid diagnostic tests. CLM data were included in the country’s 2024 TB Program Review. The inclusion of CLM data in formal M&E systems may have helped prompt national-level systems reforms, including a change in the port of entry for supplies in October 2024 which reduced the stock-out rate (71).

**Figure 3:**
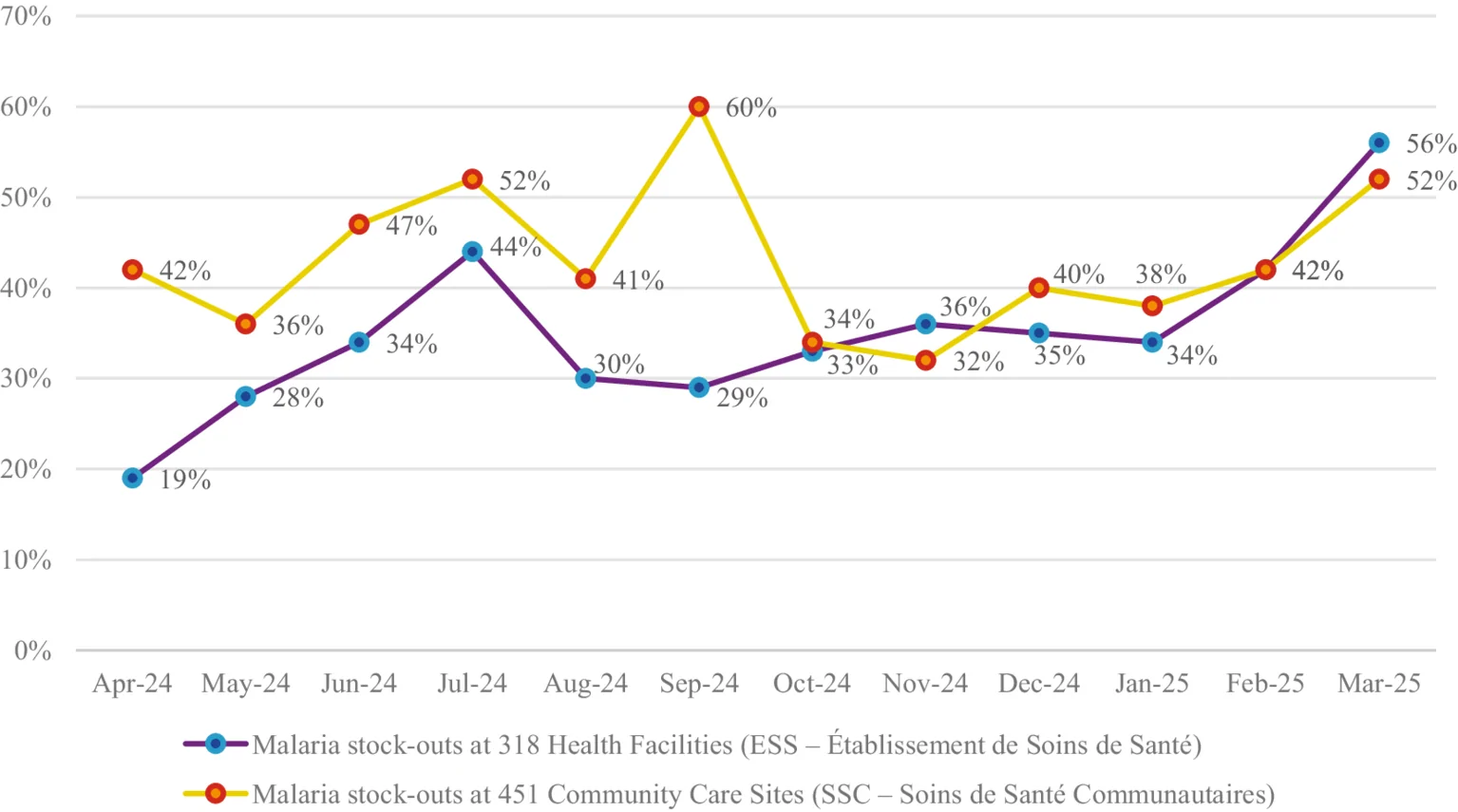
CLM data on stock-outs of malaria commodities included in the 2024 National Malaria Program Review in Democratic Republic of Congo (70).

#### 3.2.2 Integrated training, deployment and supervision of human resources for health

To maximize efficiency of the health workforce and ensure services are person-centered, it is critical that healthcare workers have the capacity and job mandate to provide integrated care (72, 73).

In Zimbabwe, CLM data collected from service users highlighted capacity gaps among service providers, including inconsistent TB screening in HIV services, gaps in malaria case management, and suboptimal client-provider interactions. These insights have informed targeted capacity building interventions, including an integrated infection prevention and control (IPC) workshop in Umzingwane which strengthened participants’ knowledge of infectious disease transmission, standard precautions, hygiene practices, and waste management. This has helped improve their ability to manage multiple infectious disease risks simultaneously, contributing to safer and more effective service delivery across HIV, TB and malaria programs. Communities also report more positive experiences interacting with healthcare workers (74).

In Lesotho, 33% (*n* = 2,383) of CLM respondents across 150 health centers felt that healthcare workers were unfriendly when providing services (75). This led to a recommendation from CLM implementers for integrated training on counseling and motivational sessions for health facility staff to address their mental health needs as provider.

#### 3.2.3 Integrated procurement and supply chain systems

Public health supply chains in low and middle-income countries often suffer from duplicated efforts and other inefficiencies from poor integration across departments, programs (HIV, TB, and malaria) and levels (central and regional) (76). An integrated public health supply chain requires a coordinated network of stakeholders, inventory systems, and forecasting models, aimed at getting health products to the point of care (76, 77).

In Namibia, there is integrated CLM of commodity stock-outs across HIV, TB, malaria, family planning, providing a holistic view of supply gaps (Figure 4) (78). The program also documented shortages of antibiotics, pain relievers, allergy medication, antihypertensives, and insulin. Finding suggest that CLM may help identify root causes such as ineffective inventory management between health facilities and the Central Medical Store (CMS). CLM implementers are advocating for the bi-electronic Pharmaceutical Information Dashboard to be fully functional in all facilities, allowing them to check CMS stock levels and monitor transport to major pharmacies (79).

**Figure 4:**
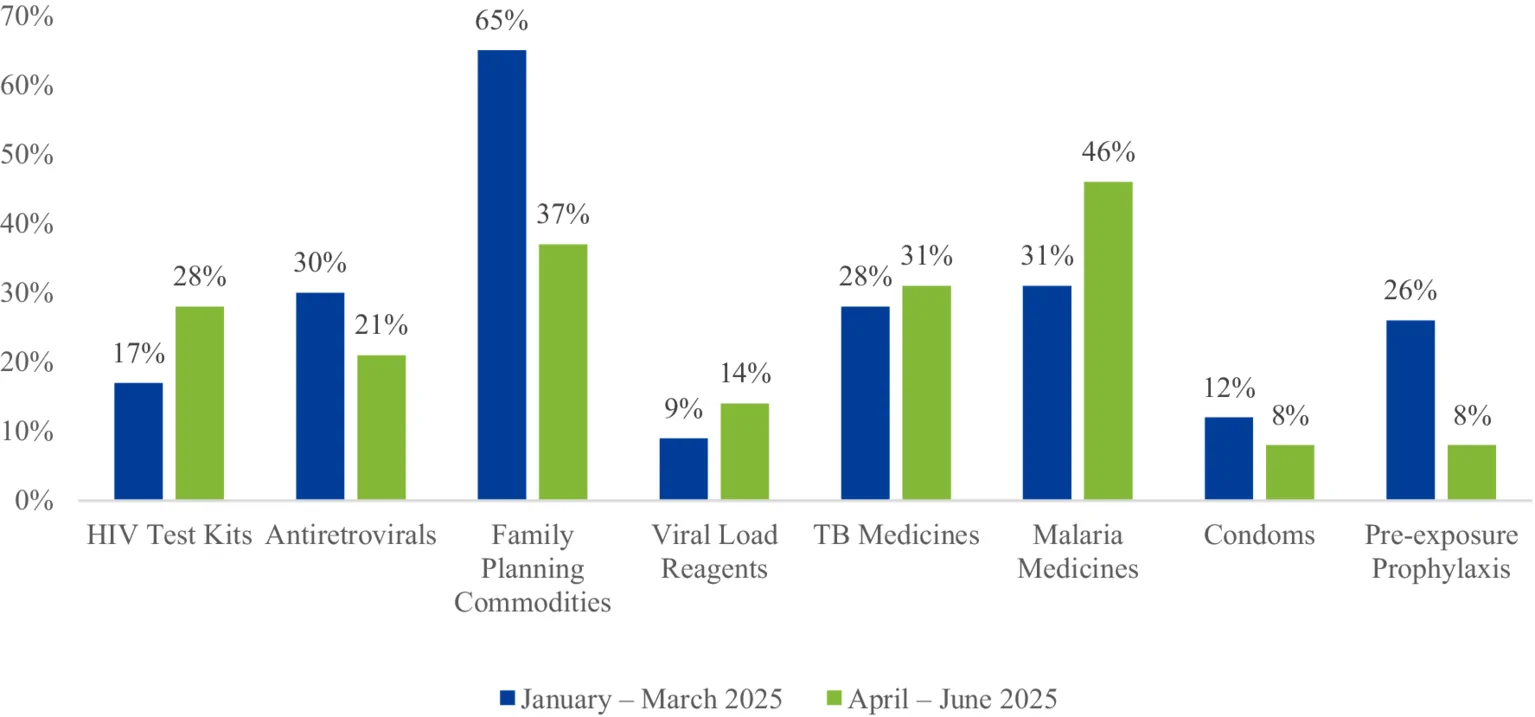
Integrated monitoring of stock-outs at 109 health facilities in Namibia, January–June 2025, CLM data (78).

#### 3.2.4 Integrated laboratory systems

A practical way to build cost-effective laboratory systems that deliver fast and reliable test results is to shift from disease-specific programs toward integrated laboratory services (80, 81). Integrated sample transportation systems have been shown to be cheaper and faster in several African settings (82–85).

In DRC, there is integrated CLM of laboratory functions for HIV and TB. In 2025, communities documented poor sample collection for early infant diagnosis, delayed return of viral load test results (with 35% never returned) and poor TB testing protocols (15% of respondents are rarely or never screened) (86). Potentially linked to advocacy from CLM implementers and other civil society partners, recruitment of transporters for the dispatching of samples was initiated. As of November 2025, the Ministry of Public Health, Hygiene, and Social Welfare reports that the new Integrated and Secure Transport of HIV and TB Samples (TISE) is now operational (87). In March 2026, the country disseminated a new Integrated Sample Transport Guide and Operational Guideline for TB and HIV specimen transport (88).

In Burkina Faso, CLM also monitors the integrated biological specimen referral system (SITEB), which was officially launched in January 2020, transporting HIV, TB and COVID-19 samples from districts to national reference labs (89). Specifically, CLM tracks the proportion of samples delivered by the national postal service (*La Poste BF*) an outsourcing strategy to improve efficiency. From January to March 2025, CLM data show that 13% of samples were transported by the postal service, 84% by healthcare workers and 3% by other means (Figure 5). CLM implementers are advocating for a greater proportion of integrated samples to be delivered by the Post Office, since trips made by healthcare workers reduces the time they can devote to patient care (90).

**Figure 5:**
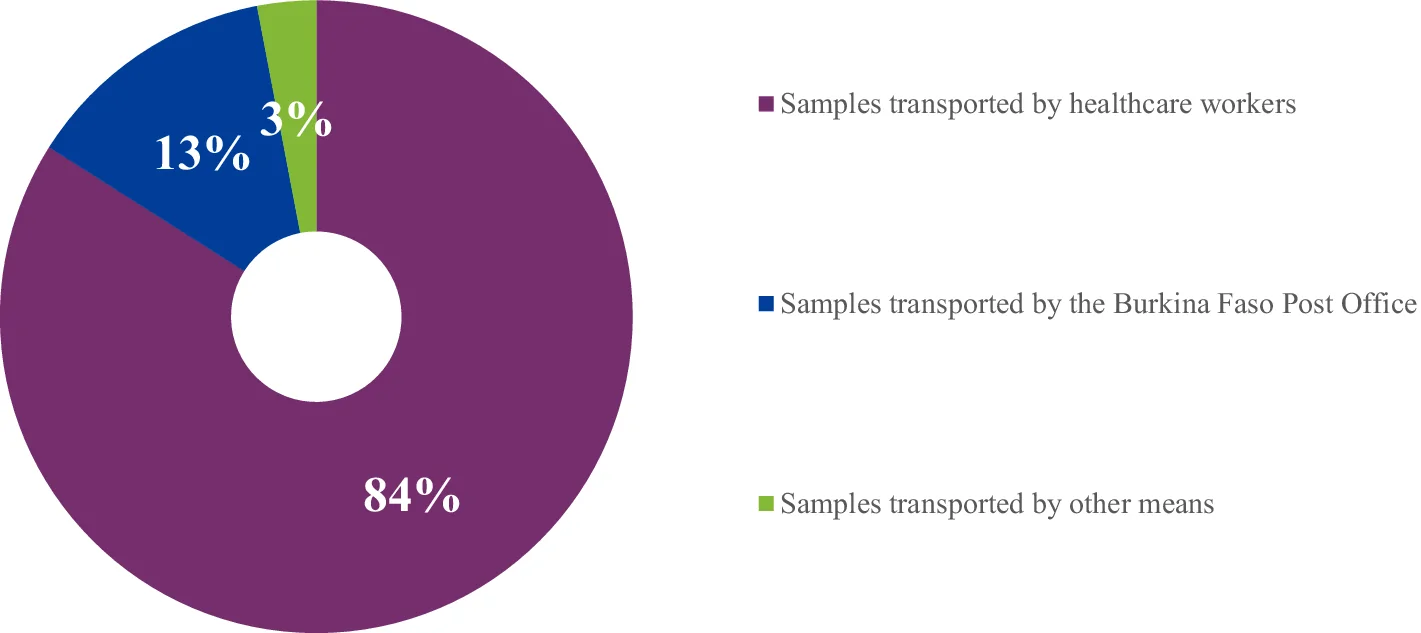
CLM data on Burkina Faso’s integrated biological specimen referral system (SITEB), 162 health facilities, January–March, 2025 (90).

In Zimbabwe, CLM tracks turnaround times for lab test results as a proxy for lab efficiency. Data collected in February 2025 in Umzingwane revealed prolonged viral load test turnaround times of 2–3 months, significantly exceeding the national target of less than 4 weeks. Additionally, only 8 out of 35 (23%) respondents knew their viral load results, highlighting gaps in result dissemination and patient follow-up. CLM identified slow, inefficient specimen transport as a root cause, and implementers advocated for integrated transportation of HIV viral load, early infant diagnosis, and TB testing samples. Integrated sample transportation in Zimbabwe has been shown to achieve an 8-day turnaround time for HIV treatment monitoring and TB sputum testing and reduce the cost per test by 73 and 86%, respectively (83).

#### 3.2.5 Integrated health financing systems

Integrated national financing frameworks help countries align and coordinate diverse funding sources to support health systems and broader sustainable development goals in a coherent manner (91). Social health insurance is a core component of integrated health financing systems, pooling contributions from multiple sources and reducing fragmentation through more coherent funding flows across health programs (92).

In Rwanda, the CLM program tracks whether access to health insurance is a barrier to health services (93). In 2023/24, 85.3% of the Rwandan population had some form of health insurance, with lower coverage among the poorest quintile (76%) (94). CLM data suggest coverage may be even lower for priority populations, with just 10% (*n* = 37/370) of young people living with HIV reporting access to insurance in January 2024 (110). CLM also found that in November 2024, more than a third of adolescents and young people report being denied health services, among whom 37% site inability to pay and insurance gaps as the reason (95). CLM implementers are advocating for aligned financing to sustain integrated youth-friendly HIV services (96). This CLM data was used in Rwanda’s HIV, TB and Malaria Sustainability Plan 2026–2030 to support expansion of community-based health insurance (CBHI) (97).

In Lesotho, the government seeks to harness CLM to improve tracking of government budget performance across sectors, including health, education, water and sanitation, and infrastructure (98).

#### 3.2.6 Integrated policy and planning that includes HIV, TB and malaria

As part of integration, countries are encouraged to move from vertical, disease-specific plans to unified, system-oriented national health strategies that consider multiple facets of information (99, 100).

In DRC, findings suggest that CLM data contributes to integrated local planning. In several Decentralized Provincial Health Divisions (DPS) priorities identified through CLM are included in community health micro plans.

In Ghana, CLM is embedded and institutionalized within community health management committees, which support and oversee the community-based health planning and services (29). The approach is called Community Engagement for Health and Wellbeing (CE4WH). Through extensive district, regional and national dialogs, and alignment with national strategies, policies, and available data, Community Health Action Plans (CHAPs) are developed (101).

In Liberia, information on CLM investments and intervention sites is published by the Ministry of Finance and Development Planning on its Development Dashboard (102). This may help support donor coordination and harmonized community responses in the country.

#### 3.2.7 Integrating community systems into broader health systems

Integrating community systems into broader health systems is essential to improve equity, extend service coverage, strengthen accountability, and ensure that health responses are comprehensive, sustainable and responsive to population needs (103–105). CLM provides a key entry point for communities to engage in formal health decision-making spaces. Integrating the Health and Community System Strengthening strategies is a core CLM program goal in Lesotho (75).

In South Africa, after CLM documented significant service gaps for people who use drugs, three members from this community—including one CLM data collector—joined the Clinic Health Committees and Hospital Board Committees in Cape Town and Ekurhuleni Districts (106). This formally integrates the community into health governance structures and may help to ensure that CLM data can further influence decision-making.

## 4 Discussion

This case study suggests that CLM can contribute to efforts to advance the integration of HIV, TB and malaria services into broader health systems, consistent with other CLM reviews (107). Data from the 16 countries reveal persistent fragmentation in service delivery and health systems, consistent with prior research highlighting the limitations of vertical, disease-specific programs (2–4). Findings in this case study confirm previous findings that this lack of integration contributes to inefficiencies, missed opportunities for co-infection management, and poorer quality of care (9, 11). At the same time, this study extends the literature by illustrating how CLM can identify integration gaps and generate evidence that is subsequently used in advocacy, planning, and other corrective processes. These findings suggest that CLM may function not only as a diagnostic tool but also as a contributor to broader reform processes.

Across the 16 countries, 13 (81%) monitored both HIV and TB, including eight (50%) that conducted integrated monitoring across HIV, TB and malaria. Fourteen (88%) monitored at least one additional health or cross-cutting issue beyond HIV, TB and malaria. Two countries documented incorporation of CLM data into national routine monitoring or review systems: Nigeria through interoperability with DHIS2 and the National Data Repository, and DRC through systematic inclusion in annual HIV, TB and malaria program reviews. Three countries—Burkina Faso, DRC and Zimbabwe—reported CLM contribution to integrated laboratory or specimen-transport systems, while at least four—DRC, Lesotho, Rwanda and South Africa—reported formal policy, planning or governance changes associated with CLM evidence. These counts are descriptive and do not imply equivalent implementation depth or direct causal attribution to CLM.

Table 2 synthesizes the main cross-country patterns influencing CLM-supported integration. Common facilitators included integrated monitoring tools, sustained advocacy, collaboration with national authorities and alignment with national planning, although priorities varied by epidemiological context, donor transition, legal and policy environments and the role of non-health sectors. Measurable evidence of implementation, scale, sustainability and direct attribution to CLM remained uneven, with the strongest progress observed where CLM data were embedded in formal monitoring, planning and governance structures.

**Table 2:** Cross-country synthesis of factors influencing CLM-supported integration.

Theme	Cross-country synthesis	Illustrative country examples
Common facilitators	Integrated CLM tools, sustained advocacy with decision-makers, collaboration with national health authorities, and alignment with national planning processes helped translate community data into action that advanced the integration agenda.	DRC, Ghana, Guinea, Nigeria, South Africa
Shared challenges and evidence gaps	CLM more consistently identified integration problems than documented measurable improvements. In several countries, advocacy or monitoring was ongoing, with limited evidence of implementation, scale, sustainability or direct attribution to CLM.	Angola, Burkina Faso, Ethiopia, Lesotho, Namibia, Nigeria
Contextual differences	Integration priorities varied according to epidemiolical context, donor transition timelines, the legal and policy environment, and the role of non-health sectors in HIV, TB and malaria responses.	Ethiopia, Liberia, Malawi, Mali, Mauritius, Rwanda, South Africa
Health system factors influencing success	Progress was strongest where CLM data were incorporated into formal monitoring, planning and governance structures. Data appear most influential where integration processes for workforce, supply chain, laboratory and financing systems were already underway.	DRC, Burkina Faso, Ghana, Nigeria, Rwanda, Zimbabwe

Although this case study focuses on Africa, studies elsewhere report similar effects, lessons and enabling conditions for CLM-supported integration. In India and Indonesia, CLM identified fragmentation between HIV and viral hepatitis services and supported stronger referral, laboratory and supply chain linkages (35). In Latin America, CLM contributed to improved diagnostic coordination, insurance access, and policy alignment (108), while evidence from Ukraine shows that integration into formal policy, financing and governance structures is critical for institutionalizing CLM and sustaining its influence (109). These parallels suggest that CLM’s role in identifying fragmentation and promoting more coordinated services, systems and governance is not specific to Africa, although the form and pace of integration remain context-dependent.

The findings are also not inherently limited to countries receiving Global Fund support. The review included documentation from both Global Fund-supported programs and those funded by other partners. The relevance of the core CLM cycle—community-led data collection, analysis, engagement and advocacy—is donor agnostic. Funding sources and the available technical assistance may nevertheless affect program scale, independence, institutionalization, and sustainability.

Several key lessons emerge from this analysis. First, the design of CLM tools is critical: integrated indicator frameworks that capture multiple diseases and system functions enable more holistic analysis and action. Second, the role of CLM implementers is central; diverse community organizations act as brokers of integration, engaging actors across health and non-health sectors, including justice systems and social services. Third, CLM may have relevance beyond service-level improvement, with the potential to contribute to systems-level reform processes by informing policy, planning, and resource allocation. Finally, in the context of declining external funding, CLM may offer a pragmatic means of identifying fragmentation within health systems and informing efforts to improve efficiency and sustainability.

These findings reinforce the growing consensus that integration—co-led with communities—is important for sustaining gains in HIV, TB and malaria responses (12, 107) and suggest that CLM may contribute to this transition by generating community evidence on service and systems fragmentation and informing responses to identified gaps.

Future research should examine how CLM can remain sustainable as health systems integrate and external funding declines. This includes testing whether CLM can be delivered through shared tools and existing structures, such as clinic health committees, while preserving community independence and representation of marginalized groups. Studies should compare the cost, effectiveness and accountability of integrated and stand-alone models.

## 5 Limitations

This study has several limitations. First, the 16 countries were purposively selected because they were priority countries receiving dedicated technical assistance for CLM, rather than sampled to be representative of African countries more broadly. The findings should therefore not be interpreted as generalizable to all African settings. Second, the underlying evidence was heterogeneous in scope, methodology, reporting periods, geographic coverage, and level of detail, which limits comparability across countries and precludes standardized assessment of the magnitude of observed effects. Third, much of the evidence was derived from reports produced by CLM implementing organizations. Although findings were triangulated with peer-reviewed literature and national health authority sources where possible, reliance on implementer-generated documentation may introduce reporting or positive-outcome bias, particularly because successful examples of advocacy or systems change may be more likely to be documented than unsuccessful or incomplete efforts. Finally, this observational case study was not designed to establish causal relationships between CLM activities and subsequent service, policy, or health-system changes. In several cases, CLM identification of a problem or advocacy activity preceded a reported change, but this alone does not establish attribution. The findings should therefore be interpreted as evidence of associations, temporal sequences, and plausible contributions of CLM rather than proof that CLM directly caused the observed changes.

Further, while not a limitation of the study, it is important to note the limitations of CLM as a intervention. CLM impact depends on the willingness and capacity of duty bearers to respond to community-generated evidence, while uneven institutionalization and continued reliance on donor funding may constrain sustainability and long-term impact (30, 74).

## Data Availability

The original contributions presented in the study are included in the article/supplementary material, further inquiries can be directed to the corresponding author.
